# Accidents of Knighthood

**Published:** 1858-05

**Authors:** 


ACCIDENTS OF KNIGHTHOOD.—We copy, without vouching for its authenticity, the subjoined paragraph from *The Press* of this city, respecting the manner in which Sir Philip Crampton, the celebrated Irish surgeon, now far advanced in years, received the honors of knighthood.

"George IV. visited Ireland in 1821, at which time Philip Crampton was Surgeon-General of the forces. He attended the King's *levée*, and wore the proper official uniform of his station, blue, with gold lace, epaulets, sword, and plumed cocked hat. This attire resembled that of a General of Artillery, and Crampton, who was over six feet high, and one of the handsomest men in Ireland at that time, looked remarkably well in it. His appearance struck the King, who made a remark to Lord Norbury, 'Fine man! General officer? in what branch of the service?' Norbury, who was a better courtier than to insinuate that royalty possibly could be mistaken, and too witty to sacrifice the opportunity of making a pun on Crampton's profession, answered, 'His name is Crampton, may it please your Majesty, and he is a General in the *Lancers*.'"

